# Identification and Biotyping of *Pythium insidiosum* Isolated from Urban and Rural Areas of Thailand by Multiplex PCR, DNA Barcode, and Proteomic Analyses

**DOI:** 10.3390/jof7040242

**Published:** 2021-03-24

**Authors:** Zin Mar Htun, Aree Laikul, Watcharapol Pathomsakulwong, Chompoonek Yurayart, Tassanee Lohnoo, Wanta Yingyong, Yothin Kumsang, Penpan Payattikul, Pattarana Sae-Chew, Thidarat Rujirawat, Paisan Jittorntam, Chalisa Jaturapaktrarak, Piriyaporn Chongtrakool, Theerapong Krajaejun

**Affiliations:** 1 Department of Pathology, Faculty of Medicine, Ramathibodi Hospital, Mahidol University, Bangkok 10400, Thailand; cutezin24528@gmail.com; 2 Department of Microbiology, Faculty of Medicine, Siriraj Hospital, Mahidol University, Bangkok 10700, Thailand; piriyaporn@gmail.com; 3 Department of Microbiology, University of Medicine, Mandalay 05024, Myanmar; 4 Department of Large Animal and Wildlife Clinical Sciences, Faculty of Veterinary Medicine, Kasetsart University, Nakhon Pathom 73140, Thailand; areelaikul@gmail.com; 5 Equine Clinic, Kasetsart University Veterinary Teaching Hospital, Nakhon Pathom 73140, Thailand; watgolf2000@gmail.com; 6 Department of Microbiology and Immunology, Faculty of Veterinary Medicine, Kasetsart University, Bangkok 10900, Thailand; fvetcny@ku.ac.th; 7 Research Center, Faculty of Medicine, Ramathibodi Hospital, Mahidol University, Bangkok 10400, Thailand; tassanee.loh@mahidol.ac.th (T.L.); wanta.yin@mahidol.ac.th (W.Y.); pusjeckchon@hotmail.com (Y.K.); payattikul@yahoo.com (P.P.); pattarana.sae@mahidol.ac.th (P.S.-C.); thidarat.ruj@mahidol.ac.th (T.R.); paisan.jit@mahidol.ac.th (P.J.); chalisa.jat@mahidol.edu (C.J.)

**Keywords:** pythiosis, *Pythium insidiosum*, environment isolate, DNA barcode, biotyping

## Abstract

*Pythium insidiosum* causes pythiosis, a fatal infectious disease of humans and animals worldwide. Prompt diagnosis and treatment are essential to improve the clinical outcome of pythiosis. Diagnosis of *P. insidiosum* relies on immunological, molecular, and proteomic assays. The main treatment of pythiosis aims to surgically remove all affected tissue to prevent recurrent infection. Due to the marked increase in case reports, pythiosis has become a public health concern. Thailand is an endemic area of human pythiosis. To obtain a complete picture of how the pathogen circulates in the environment, we surveyed the presence of *P. insidiosum* in urban (Bangkok) and rural areas of Thailand. We employed the hair-baiting technique to screen for *P. insidiosum* in 500 water samples. Twenty-seven culture-positive samples were identified as *P. insidiosum* by multiplex PCR, multi-DNA barcode (rDNA, *cox*1, *cox*2), and mass spectrometric analyses. These environmental strains of *P. insidiosum* fell into Clade-II and -III genotypes and exhibited a close phylogenetic/proteomic relationship with Thai clinical strains. Biodiversity of the environmental strains also existed in a local habitat. In conclusion, *P. insidiosum* is widespread in Thailand. A better understanding of the ecological niche of *P. insidiosum* could lead to the effective prevention and control of this pathogen.

## 1. Introduction

*Pythium insidiosum* is a unique pathogenic oomycete that causes the devastating infectious condition, termed “pythiosis”, predominantly in humans, horses, and dogs [1,2,3,4]. The disease is prevalent in tropical and subtropical countries. Affected individuals usually present with an infection of the skin, eye, artery, or internal organ [1,2,3,4]. The pathogenesis mechanism of *P. insidiosum* has begun to be better understood through genomic and transcriptomic analyses [5,6,7,8,9,10,11,12]. Pythiosis possesses a high morbidity and mortality rate, as it is difficult to diagnose and treat the disease. Early diagnosis and prompt treatment are essential to improve the clinical outcome of a pythiosis patient. The definitive diagnosis of *P. insidiosum* cannot rely on the microbiological findings (i.e., colony morphology and zoospore), but requires an immunological [i.e., immunodiffusion, enzyme-linked immunosorbent assay (ELISA), and immunochromatography], molecular (i.e., sequence homology, polymerase chain reaction (PCR), and loop-mediated isothermal amplification (LAMP), or proteomic [i.e., matrix-assisted laser desorption/ionization time-of-flight mass spectrometer (MALDI-TOF MS)] assay [3,4,13,14,15,16,17,18,19,20,21,22,23,24,25]. Such the diagnostic tests are not widely available in the clinical laboratories, leading to the delayed diagnosis. Although the antifungal drugs are generally ineffective against the *P. insidiosum* infection due to the lack of drug-target ergosterol biosynthesis enzymes, success stories of antimicrobial drug use have been occasionally reported [2,4,26,27]. In most cases, the treatment of pythiosis aims to surgically remove all infected tissue [1,2,3,4]. However, a post-surgical recurrent infection is not uncommon due to residual infected tissues [2,4]. Vaccine immunotherapy has been used in the treatment of pythiosis, but it shows limited efficacy [2,4].

Thailand is an endemic area of human pythiosis, and the most affected individuals are farmers [2,4]. Human pythiosis is associated with several hematological disorders (especially thalassemia), in which the underlying mechanism is unknown [2,4,28]. Several case series of human pythiosis (mainly ocular infection) were recently reported from India [29,30,31]. Pythiosis in animals (i.e., horses and dogs) has been mostly diagnosed in other countries, especially Brazil and the United States [1,3,32]. Due to the marked increase in case reports, pythiosis has become a public health concern. As a part of its life cycle, *P. insidiosum* produces an infective unit, called biflagellate zoospore, in water [33]. Plant materials and animal hairs can attract the organism [34,35,36]. When a swimming zoospore comes in direct contact with an individual, it germinates as hyphae and causes tissue pathology [33,34]. Better understanding the ecological niche of *P. insidiosum* could lead to the effective prevention and control of this pathogen, especially for those individuals at risk.

Several investigators can isolate *P. insidiosum* from agricultural or non-residential areas in northern Thailand, Australia, the United States, and Brazil [34,35,36,37,38,39]. To obtain a complete picture of how the pathogen circulates in the environment, we aim to survey the presence of *P. insidiosum* in a crowded city like Bangkok, as well as rural areas of central and southern Thailand. We employed the hair-baiting technique to isolate *P. insidiosum* from 500 water samples. Colony morphology was used as a high-throughput screening method, and the culture-positive samples were confirmed by several molecular assays, including multiplex PCR, multi-DNA barcode, and proteomic analyses [4,19,23,40,41,42,43]. We successfully isolated *P. insidiosum* from Bangkok and other provinces, in which some areas had a notably-high prevalence of the organism. We explored biodiversity, proteomic feature, and phylogenetic relationship of the environmental and clinical strains of *P. insidiosum* and proposed the use of multi-DNA barcodes for the identification of this pathogen.

## 2. Materials and Methods

### 2.1. Sample Collection and Culture Condition

A total of 500 water samples were collected from 100 sample-collection sites in 23 sampling locations (i.e., zoo, public parks, rice fields, and ponds) across 7 provinces of Thailand, which included Bangkok (10 locations; 48 sites; 240 samples), Chonburi (1 location; 3 sites; 15 samples), Chachoengsao (3 locations; 8 sites; 40 samples), Nakhon Pathom (1 location; 12 sites; 60 samples), Kanchanaburi (3 locations; 11 sites; 55 samples), Ratchaburi (4 locations; 13 sites; 65 samples), and Trang (1 location; 5 sites; 25 samples) (Table 1). Five water samples (500 mL/sample) were collected from each sample-collection site using a clean disposable plastic bucket (sampling position: 50–100 cm away from the bank; 5–10 cm depth from the water surface). Each water sample was transferred to a sterile plastic bag containing 5–10 autoclaved 10-cm-long human hairs and left at the ambient temperature overnight. The hairs were removed from the bag by sterile forceps and incubated on Sabouraud dextrose agar (pH 7.2) supplemented with penicillin and streptomycin (100 µg/mL each; Sigma-Aldrich, St. Louis, MO, USA) at 25 °C for 5 days. A growing, submerged, white-to-colorless colony, which is compatible with *P. insidiosum*, was subculture onto a freshly-prepared Sabouraud dextrose agar (with or without an overlayed sterile cellophane membrane) and subject to the downstream genomic DNA (gDNA) extraction.

### 2.2. Genomic DNA Extraction

Up to 200 mg of an obtained colony were harvested for gDNA extraction by adapting the salt extraction protocol described by Lohnoo et al. [21]. A hyphal mat was transferred to a 2-mL sterile plastic screw-cap tube containing 1000 mg of glass beads (710–1180 mm in diameter; Sigma, St. Louis, MO, USA) and combined with the salt homogenizing buffer (0.4 M NaCl, 10 mM Tris-HCl pH 8.0, and 2 mM EDTA; 400 μL buffer per 100 mg hyphae). To remove carry-over culture agar from the harvested hyphae, the sample tube was boiled at 100 °C for 5 min. The hyphal mat was ruptured by a Tissue Lyzer Retsch MM301 (setting: 2 min at 30 Hz; Qiagen, Hilden, Germany) and mixed with 45 μL of 20% SDS and 8 μL of 20 mg/mL proteinase K, before an overnight incubation at 56 °C. After well-mixed with 0.3 mL of 6 M NaCl, the sample was centrifuged at 10,000× *g* for 30 min. The supernatant was collected, combined with an equal volume of isopropanol, stored at −20 °C for 1 h, and centrifuged (10,000× *g*) at 4 °C for 20 min. After discarding the supernatant, a resulting pellet was collected, washed with 70% ethanol, air dried, and resuspended in 100 μL of Tris-EDTA (10 mM Tris, 1 mM EDTA; pH 8.0). A NanoDrop 2000 spectrophotometer estimated DNA concentration at 260/280 nm wavelengths (Thermo Scientific, Waltham, MA, USA).

### 2.3. Multiplex PCR for Identification and Genotyping of P. insidiosum

The established multiplex PCR assay targeting the rDNA sequence was used to identify and genotype *P. insidiosum* [23]. A 25-μL PCR reaction comprised 50 ng of gDNA template, 0.1 µM of the primer ITS1 (5′-TCCGTAGGTGAACCTGCGG-3’), 0.07 µM each of the primers R1 (5′-CCTCACATTCTGCCATCTCG-3′), R2 (5′-ATACCGCCAATAGAGGTCAT-3′) and R3 (5′-TTACCCGAAGGCGTCAAAGA-3′), 0.2 mM dNTP, 2 mM MgCl_2_, 0.65 U *Taq* polymerase (Thermo Scientific), and 1× buffer with KCl. The reaction was carried out with the following thermal cycling condition: the initial 95 °C denaturation for 5 min, 30 cycles of 95 °C denaturation for 30 s, 59 °C annealing for 30 s and 72 °C extension for 45 s, and the final 72 °C extension for 10 min. The resulting amplicons were assessed for amplicon sizes, using the capillary electrophoresis-based QIAxcel advanced system, DNA screening kit (method AM320), 15–5000 bp alignment markers, and QIAxcel screen gel software (Qiagen). The presence of the 490- and 660-bp (Clade-I genotype), 660-bp (Clade-II genotype), or 800-bp (Clade-III genotype) band(s) indicates *P. insidiosum*.

### 2.4. Species Identification by DNA Barcode Analysis

All extracted gDNA samples were recruited for species identification using the rDNA sequence (i.e., the ITS1–5.8S-ITS2 region) as the primary DNA barcode. PCR amplification was carried out in a 50-μL reaction containing 50 ng gDNA template, the universal fungal primers [0.1 µM each of ITS1 and ITS4 (5′-TCCTCCGCTTATTGATATGC-3′)] [40], 0.2 mM dNTP, 2 mM MgCl_2_, 0.65 U *Taq* polymerase (Thermo Scientific), and 1× buffer with KCl. The thermal cycle program included the initial 95 °C denaturation for 5 min, 30 cycles of 95 °C denaturation for 30 s, 55 °C annealing for 30 s, and 72 °C extension for 30 s, and the final 72 °C extension for 10 min.

The secondary DNA barcode sequences (i.e., *cox*1 and *cox*2) were also amplified from gDNA samples of *P. insidiosum* and another *Pythium* spp. A PCR amplification was set up in a 50-μL reaction, containing 100 ng gDNA template, the oomycete-specific *cox*1 primers [0.2 μM each of OomCox-I_Levup (5′-TCAWCWMGATGGCTTTTTTCAAC-3′) and OomCox-I_Levlo (5′-CYTCHGGRTGWCCRAAAAACCAAA-3′)] [42] or *cox*2 primers [0.4 μM each of FM58 (5′-CCACAAATTTCACTACATTGA-3′) and FM66 (5′-TAGGATTTCAAGATCCTGC-3′)] [41], 0.2 mM dNTP, 1.5 mM MgCl_2_, 1.25 U *Taq* DNA polymerase (Thermo Scientific), and 1× buffer with KCl. The thermal cycle settings included the initial 94 °C denaturation for 5 min, 30 (*cox*1) or 35 (*cox*2) cycles of 94 °C denaturation for 30 (*cox*1) or 60 (*cox*2) s, 52 °C annealing for 30 (*cox*1) or 60 (*cox*2) s, and 72 °C extension for 45 (*cox*1) or 60 (*cox*2) s, and the final extension at 72 °C for 10 min.

All PCR products were checked using the QIAxcel advanced system (as mentioned above). The PCR products were cleaned using a PCR purification kit (Qiagen) and sequenced using a corresponding primer set, such as ITS1/ITS4 (rDNA), OomCox-I_Levup/OomCox-I_Levlo (*cox*1), and FM58/FM66 (*cox*2). The obtained sequence was BLAST searched against the NCBI nucleotide database (https://blast.ncbi.nlm.nih.gov/Blast.cgi accessed on 16 January 2021). The BLAST search cutoff for species identification was set at 98.5% identity [43].

### 2.5. Phylogenetic Analysis

The obtained rDNA, *cox*1, and *cox*2 sequences of each of the *P. insidiosum* strains and outgroup organisms were concatenated and subject to phylogenetic analysis, using the online bioinformatics tool at (http://www.phylogeny.fr accessed on 16 January 2021) [44]. The “one-click” default setting [44] executed multiple sequence alignment using MUSCLE (v3.8.31) [45], sequence curation using GBlocks (v0.91b) [46], phylogenetic relationship analysis using PhyML (v3.0) [47] and branch assessment using a LRT test [48], and phylogenetic tree construction using TreeDyn (v198.3) [49].

### 2.6. Mass Spectrometric Analysis and Dendrogram

Selected organisms (i.e., a colony without bacterial contamination) isolated from the environment (i.e., *P. insidiosum*, *Pythium catenulatum*, and *Pythium rhizo-oryzae*) (Table 1 and Table 2), *P. insidiosum* strain Pi35 isolated from a patient with pythiosis (positive control) and *Candida parapsilosis* strain ATCC 22019 (negative control) were recruited for MALDI-TOF MS analysis. Each organism was subcultured in a glass Petri dish containing 10 mL Sabouraud dextrose broth and incubated at 37 °C for 5–7 days. Proteins were extracted from these organisms using the established protocol [19] with some modifications. Briefly, a harvested organism (100 mg) was transferred to a sterile microtube, washed 5 times with 1 mL of liquid chromatography-mass spectrometry (LC-MS) grade water (Merck, Kenilworth, NJ, USA), and centrifuged (16,600× *g*) at 4 °C for 5 min to remove the supernatant. The organism was mixed with 300 µL of LC-MS grade water before vortexing and adding 900 µL of absolute EtOH (Merck). The sample mixture underwent another round of vortexing, centrifugation, and supernatant removal. A resulting pellet was dried at 37 °C for 30 min and resuspended with equal volumes (up to 100 µL) of 70% formic acid (Merck) and acetonitrile (Merck). The supernatant (containing extracted proteins) was collected by centrifugation and kept at −30 °C until use.

The extracted protein (0.5 µL) was placed on a ground steel target plate (Bruker Daltonics, Billerica, MA, USA) (8 replicates), air-dried, and layered with 0.5 µL of 5 mg/mL α-cyano-4-hydroxycinnamic acid in 70% acetonitrile and 0.1% trifluoroacetic acid. An ultrafleXtreme mass spectrometer and the FlexControl software version 3.0 (Bruker Daltonics), using the previously-described setting [19], generated mass spectra from the extracted proteins. The MALDI-TOF MS analysis matched the generated mass spectra of each organism against the supplemented Bruker MALDI Biotyper database DB4613 (Bruker Daltonics), containing the main spectral profiles (MSP) of 4274 bacteria, 331 fungi, 7 archaea, 1 green alga, and 13 *P. insidiosum* strains [19]. Mass spectrum similarity was transformed into an identification score by the MALDI Biotyper software version 3.0 (Bruker Daltonics). A score of 2.00 or higher indicates a reliable species-level identification, while that fall between 1.70–1.99 indicates a reliable genus-level identification. A lower score (<1.70) means an unreliable organism identification. The MATLAB software version 7.1 (MathWorks, Natick, MA, USA) generated a dendrogram of all *P. insidiosum* isolates tested, by using the distance values (for each pair of *P. insidiosum* MSPs) calculated by the MALDI Biotyper software [19].

### 2.7. Data Availability

The rDNA, *cox*1, and *cox*2 sequences of the *P. insidiosum* strains used in this study have been deposited in the GenBank/DDBJ databases (see the accession numbers in Table 2 and Table 3). The rDNA, *cox*1, and *cox*2 sequences of the *P. rhizo-oryzae* strain RCB01 (accessions: LC556053, LC553639, and LC553641, respectively) and the *P. catenulatum* strain RM9-06 (accessions: LC556067, LC553640, and LC553642, respectively), used as an outgroup in the phylogenetic analysis, have been deposited in the same databases.

## 3. Results

### 3.1. Screening the Colony Morphology of P. insidiosum Isolated from Water Samples

**Figure 1:**
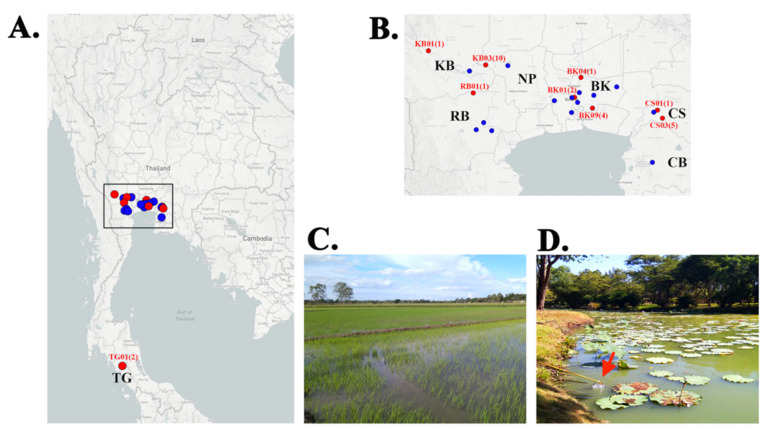
Geographic distribution of the *P. insidiosum*-positive locations in Thailand. (**A**) The map of Thailand shows 23 sampling locations (i.e., zoo, public parks, rice fields, and ponds) across 7 central and southern provinces of Thailand, which include Bangkok (BK; 10 locations: BK01-10), Chonburi (CB; 1 location: CB01), Chachoengsao (CS; 3 locations: CS01-03), Nakhon Pathom (NP; 1 location: NP01), Kanchanaburi (KB; 3 locations: KB01-03), Ratchaburi (RB; 4 locations: RB01-04), and Trang (TG; 1 location: TG01). (**B**) An enlarged map demonstrates the sampling locations shown in the box in Figure 1A. The *P. insidiosum*-positive locations (i.e., KB01, BK01, and CS01) are indicated in red. The number in each parenthesis is the number of *P. insidiosum* strain(s) successfully isolated from the corresponding location. (**C**) The location KB03 is a rice field, where 10 strains of *P. insidiosum* (strain IDs: KCB01–CB10) have been isolated. (**D**) The location BK09 is a pond in a public park in Bangkok, in which 4 strains of *P. insidiosum* (strain IDs: RM9-02–RM9-05) have been recovered (the arrow indicates a clean disposable plastic bucket used to collect water sample).

Water samples were collected from 100 water collection sites [i.e., rice fields, irrigation channels, and ponds (in a zoo, public recreation parks, and countryside areas); 5 samples/site] in 10 urban (i.e., Bangkok) and 13 rural (i.e., 5 central and 1 southern provinces) sampling locations (Table 1). All 23 locations were depicted in Figure 1 (also available online at https://microreact.org/project/nv2faGXa2rahFjUHKd5QQN access on 16 January 2021 [50]). Human hairs, used to bait *P. insidiosum* in a water sample, were incubated at room temperature on a Sabouraud agar plate supplemented with the antibacterial agents. From the total of 500 water samples, 446 (89.2%) showed different bacterial growths and various fungal colonies (with or without spores or color pigments), while 54 (10.8%) exhibited no growth on the agar plates. Among them, 64 samples (12.8%) provided a white-to-colorless, non-sporulation, submerged colony, which is compatible with the gross morphology of *P. insidiosum*. Each suspected *P. insidiosum* colony was subcultured on a new Sabouraud agar plate for gDNA extraction. The obtained gDNA samples were used for species identification, biotyping, and phylogenetic analysis (see below).

### 3.2. Identification and Genotyping of P. insidiosum by Multiplex PCR and DNA Barcodes

**Figure 2:**
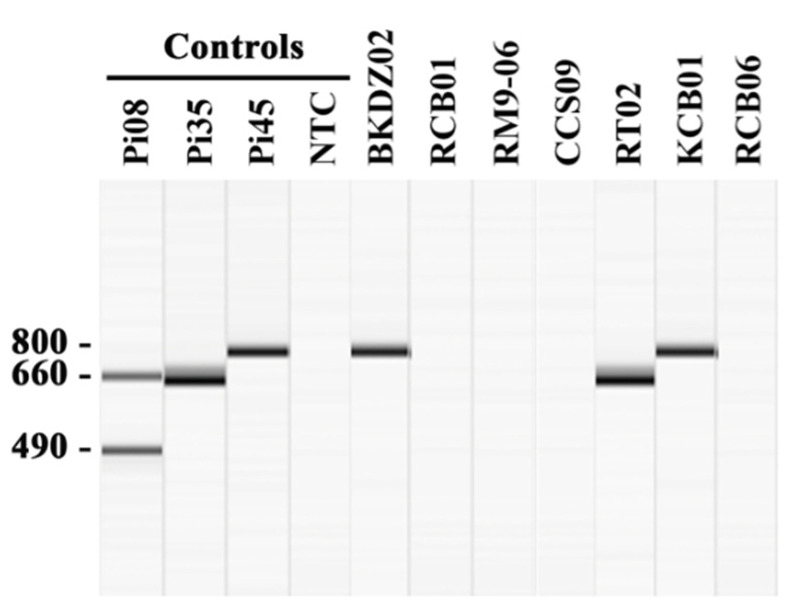
Identification and genotyping of *P. insidiosum* by multiplex PCR. The multiplex PCR amplifies the rDNA sequence from a gDNA sample extracted from water-isolated *P. insidiosum*-suspected colonies. The amplicon sizes are assessed by using the capillary electrophoresis-based QIAxcel advanced system (Qiagen) (see the methods). The positive controls include gDNA samples extracted from *P. insidiosum* strains Pi08 (Clade-I genotype; amplicons: 490- and 660-bp bands), Pi35 (Clade-II genotype; amplicon: 660-bp band), and Pi45 (Clade-III genotype; amplicon: 800-bp band). The PCR reaction with no gDNA template serves as the negative control [no template control (NTC)]. The multiplex PCR results of 7 randomly-selected *P. insidiosum*-suspected organisms (IDs: BKDZ02, RCB01, RM9-06, CCS09, RT02, KCB01, and RCB06) are shown in the Figure.

All 64 extracted gDNA samples were initially analyzed by the established *P. insidiosum*-specific multiplex PCR [23]. The assay can identify and genotype *P. insidiosum* in 27 samples (Table 2): 70.4% (*n* = 19) of which provided only the 660-bp band (Clade-II genotype), such as the sample RT02, while the rest, 29.6% (*n* = 8), provided only the 800-bp band (Clade-III genotype), such as the samples BKDZ02 and KCB01 (Figure 2). The other 37 gDNA samples (i.e., RCB01, RM9-06, CCS-09, and RCB06) provided no PCR product (Figure 2). The species identifications of all samples were then performed by DNA barcode analysis (see below).

The fungal universal primers ITS1 and ITS4 [40] amplified the primary DNA barcode (rDNA sequence) from all 64 gDNA samples (Table 4). The sequence homology analysis using BLAST search against the GenBank database showed that the best-matched organism was *P. insidiosum* in 27 samples (5.4% of all 500 water samples), *Pythium catenulatum* in 16 samples (3.2%), *Pythium rhizo-oryzae* in 8 samples (1.6%), *Pythium inflatum* in 1 sample (0.2%), unspecified *Pythium* species in 5 samples (1.0%), and a fungus (i.e., *Sclerotium hydrophilum*, *Mucor amphibiorum*, and *Pezizomycetes* species) in 3 samples (0.6%) (Table 1). The gDNA samples of 4 organisms (Strain IDs: RT01, CCS06, KCB13, and KCB14; Table 4) repeatedly reported poor-quality sequencing chromatograms and were excluded from the BLAST search analysis. The rDNA sequences of the 27 water-isolated *P. insidiosum* strains (average length: 850 bp; range: 639-909 bp) showed the average sequence identity of 99.7% (range: 98.0–100.0%) (Table 2). One of these strains (ID: BKDZ02) best matched *P. insidiosum* at 98.0% identity which was slightly lower than the species-level cutoff value (98.5% identity) [43].

Two secondary DNA barcodes (i.e., *cox*1 and *cox*2) were employed for the *P. insidiosum* identification. The *P. insidiosum* strain KCB12 was lost, and thus excluded from the *cox*1 and *cox*2 barcoding analysis. Two primer pairs (i.e., OomCox-I_Levup/OomCox-I_Levlo [42] and FM58/FM66 [41]) respectively amplified a partial coding sequence of *cox*1 and *cox*2 from 26 water-isolated *P. insidiosum* strains (Table 2). The average sequence lengths of *cox*1 and *cox*2 were 690 bp (range: 656–696 bp) and 585 bp (range: 555–586 bp), respectively. BLAST search against the GenBank database best matched *P. insidiosum* in all 26 samples, with the average sequence identity of 98.1% (range: 94.1–100.0%) for *cox*1 and 100.0% for *cox*2 (Table 2). The percent identities of the *cox*1 sequences from 8 *P. insidiosum* strains (i.e., BKDZ01, BKDZ02, KCB01, KCB03, KCB04, KCB06, KCB08, and KCB09; mean, 94.3%; range: 94.1–94.4%) fell below the species-level cutoff value (98.5%) (Table 2).

### 3.3. Geographic Distribution of the P. insidiosum-Positive Water Samples

Twenty-seven *P. insidiosum*-positive water samples were derived from 9 sampling locations (39.1% of all 23 locations), covering 12 water collection sites (12.0% of all 100 sites), including 4 ponds in a zoo and two public parks (25.9% of all positive samples) in Bangkok metropolis, and 4 ponds (14.8%) and 4 rice fields (59.3%) in 4 countryside provinces (i.e., Kanchanaburi, Chachoengsao, Trang, and Ratchaburi) (Table 1 and Table 2; Figure 1). No *P. insidiosum*-positive samples were obtained from 2 provinces: Chonburi and Nakhon Pathom. Some *P. insidiosum* strains were co-isolated from the same water collection site, for example, 10 strains (IDs: KCB01–KCB10) from the Rice field#3 in Kanchanaburi (Figure 1C), 4 strains (IDs: RM9-02–RM9-05) from the Pond#4 in Bangkok (Figure 1D), and 3 strains (IDs: CCS03–CCS05) from the Rice field#1, and 2 strains (IDs: CCS07 and CCS08) from the Rice field#2 in Chachangsao (Table 2).

### 3.4. Phylogenetic Relationship among the Water-Isolated and Clinical Strains of P. insidiosum

**Figure 3:**
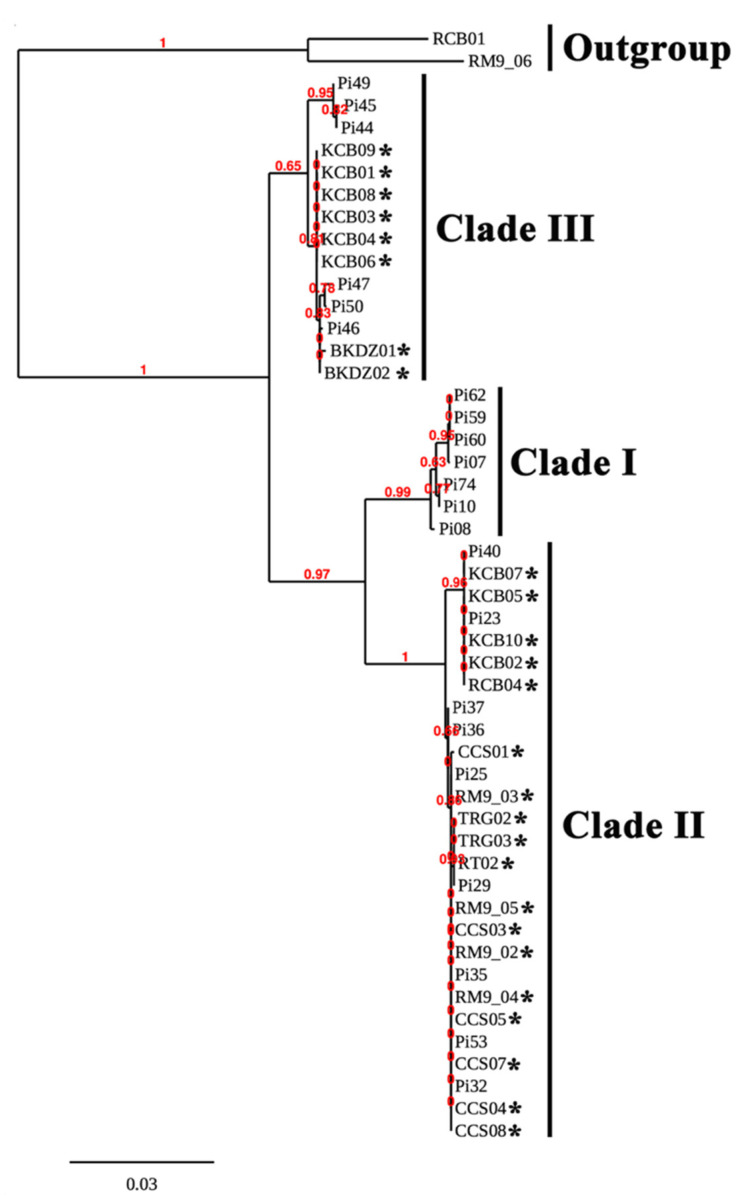
Phylogenetic relationship of water-isolated and clinical strains of *P. insidiosum*. The rDNA-*cox*1-*cox*2 concatenated sequences of 26 water-isolated (Table 2) and 22 clinical (Table 3) strains of *P. insidiosum* and 2 outgroup organisms (i.e., *P. rhizo-oryzae* strain RCB01 and *P. catenulatum* strain RM9-06) are recruited for the construction of a maximum likelihood-based phylogenetic tree. The branch support values are calculated based on the aLRT test. Asterisks indicate water-isolated strains of *P. insidiosum*.

The rDNA, *cox*1, and *cox*2 sequences of 26 water-isolated (Table 2) and 22 reference (Table 3) strains of *P. insidiosum* and 2 outgroup organisms (i.e., *P. rhizo-oryzae* strain RCB01 and *P. catenulatum* strain RM9-06) were aligned, trimmed, and concatenated into a 1737 bp-long sequence. These rDNA-*cox*1-*cox*2 combined sequences were subject to the construction of a maximum likelihood-based phylogenetic tree (Figure 3). The branch support values were calculated based on the LRT test [48]. The phylogenetic tree divided 48 strains of *P. insidiosum* into 3 groups: Clade-I, Clade-II, and Clade-III (Figure 3; Table 2 and Table 3). The water-isolated *P. insidiosum* strains only located in Clade-II (*n* = 18) and Clade-III (*n* = 8) genotypes (Figure 3; Table 2). Water from several sample collection sites (i.e., Pond#4, Rice Field#1, Rice Field#2, and Rice Field#3) contained more than one strain of *P. insidiosum* (Table 2). All 4 *P. insidiosum* strains (i.e., RM9-02, RM9-03, RM9-04, and RM9-05) isolated from Pond#3 in Bangkok, and 5 *P. insidiosum* strains isolated from Rice Field#1 (i.e., CCS03, CCS04 and CCS05) and Rice Field#2 (i.e., CCS07 and CCS08) in Chachoengsao province were grouped in Clade-II genotype (Figure 3). On the other hand, 10 *P. insidiosum* strains isolated from Rice Field#3 in Kanchanaburi province were assigned to both Clade-II (i.e., strains KCB02, KCB05, KCB07 and KCB10) and Clade-III (i.e., strains KCB01, KCB03, KCB04, KCB06, KCB08 and KCB09) genotypes (Figure 3).

### 3.5. Mass Spectrometric Analysis and Proteotyping of P. insidiosum

**Figure 4:**
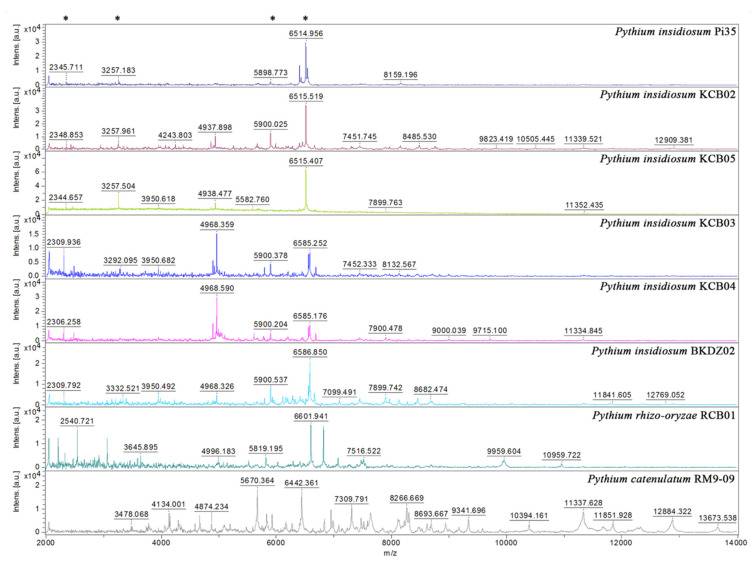
Comparison of mass spectra from *P. insidiosum* and non-*insidiosum Pythium* species. Four mass spectra are generated from *P. insidiosum* strains inhabited in the same rice field (IDs: KCB02, KCB03, KCB04, and KCB05). One each of the mass spectra is derived from *P. insidiosum* strain BKDZ02 (from a zoo in Bangkok), *P. rhizo-oryzae* strain RCB01 (from a pond in Ratchaburi province), and *P. catenulatum* strain RM9-09 (from a pond in Bangkok). The *P. insidiosum* strain Pi35 (from a pythiosis patient) is included as a reference organism. The *Y*-axis shows mass intensity, while the *X*-axis represents the mass-to-charge ratio (m/z). Asterisks indicate the prominent m/z peaks that share among different strains of *P. insidiosum.*

MALDI-TOF MS generated mass spectra from 10 selected water-isolated *P. insidiosum* strains (9 from Rice Field#3 and one from the zoo; Table 2), 2 non-*insidiosum Pythium* species (i.e., *P. rhizo-oryzae* strain RCB01 and *P. catenulatum* strain RM9-09), the *P. insidiosum* strain Pi35 (positive control), and the *C. parapsilosis* strain ATCC 22019 (negative control) (Figure 4). The obtained mass spectra were subject to the species-level identification by searching through the supplemented MALDI Biotyper DB4613 database (Bruker Daltonics), containing 4626 MSPs of various organisms, including 331 fungi and 13 *P. insidiosum* strains [19]. The mass spectrometric analysis assigned all organisms as “*P. insidiosum*” (identification scores: 2.149–2.490), except *P. rhizo-oryzae* strain RCB01 (score: 1.573), *P. catenulatum* strain RM9-09 (score: 1.483) (Table 2), and the negative control (which matched the *C. parapsilosis* strain ATCC 22019 THL; score: 2.214).

**Figure 5:**
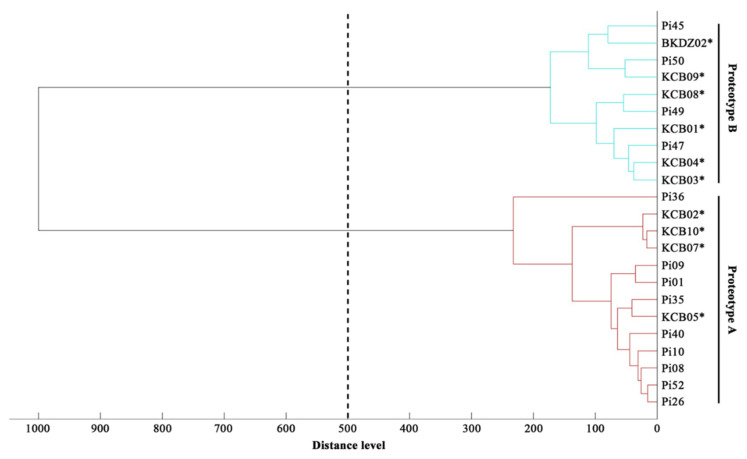
Proteomic dendrogram of water-isolated and clinical strains of *P. insidiosum*. The main spectral profiles (MSP) of 10 water-isolated (4 Clade-II and 6 Clade-III genotype strains; Table 2) and 13 clinical (4 Clade-I, 5 Clade-II, and 4 Clade-III genotype strains; Table 3) strains of *P. insidiosum* are recruited for the construction of a dendrogram. The distance value of 500 is used as the cut-off value for proteotyping of the organisms. Asterisks indicate water-isolated strains of *P. insidiosum.*

The MALDI Biotyper software constructed a dendrogram based on the MSPs of 10 water-isolated *P. insidiosum* strains (4 Clade-II and 6 Clade-III genotype strains; Table 2) and 13 *P. insidisoum* reference strains from the previous study [4 Clade-I genotype strains (i.e., Pi01, Pi08, Pi09, and Pi10); 5 Clade-II genotype strains (i.e., Pi26, Pi35, Pi36, Pi40, and Pi52); and 4 Clade-III genotype strains (i.e., Pi45, Pi47, Pi49, and Pi50)] [19]. By using the distance value of 500 as the cut-off value [51], the dendrogram divided *P. insidisoum* into 2 groups: Proteotype-A (including all Clade-I and -II genotype strains) and Proteotype-B (including all Clade-III genotype strains) (Table 2 and Table 3; Figure 5).

## 4. Discussion

We surveyed the presence of *P. insidiosum* in urban and rural watery areas of Thailand, using the hair-baiting technique [35]. A total of 500 water samples were collected from 100 sites (i.e., ponds and rice fields) in 7 central and southern provinces of the country (Figure 1). Cultures of most water samples (89.2%) showed growing bacterial and fungal colonies. Among them, 64 samples (12.8%) provided a white-to-colorless, non-sporulation, submerged colony, which is compatible with the gross morphology of *P. insidiosum*. Such morphologies are not specific to *P. insidiosum*, as they are observed in several microorganisms. Nevertheless, recognition of the colony characteristics can facilitate the screening of *P. insidiosum* through a vast number of water samples. Multiplex PCR [23] identified *P. insidiosum* in 27 out of 64 colony screening-positive samples (Table 2 and Table 4). The identity of *P. insidiosum* was confirmed by DNA barcode analyses (i.e., rDNA, *cox*1, and *cox*2) [40,41,42,43]. rDNA is the most common barcode used to identify an organism at the species level [40,43]. However, rDNA failed to assign *P. insidiosum* in one of 27 PCR-positive samples (sequence identity cutoff: 98.5%) (Table 2), indicating that the current rDNA database had a limitation in identifying this organism. The secondary barcodes (*cox*1 and *cox*2) were then employed [41,42,43]. *cox*2 identified *P. insidiosum* in all 27 PCR-positive samples (sequence identity: 100%), whereas *cox*1 detected the organism only in 19 PCR-positive samples (sequence identity: 99.3–100.0%) (Table 2). The ineffectiveness of *cox*1 in the identification of *P. insidiosum* was due to the limited *cox*1 database in GenBank, as only 4 *cox*1 sequences of this species (accessions: JQ305799, HQ708612, HQ708611, and AP014838) were available at the time of analysis. As the final result, the colony screening, multiplex PCR, and DNA barcode analyses co-identified *P. insidiosum* in 27 out of 500 water samples (detection rate: 5.4%) (Table 2).

*P. insidiosum* can be isolated from swampy areas in several countries across the world (i.e., Thailand, Australia, the United States, and Brazil) [34,35,36,37,38,39]. Recently, Jara et al. successfully isolated *P. insidiosum* throughout the study area in the Chincoteague National Wildlife Refuge in Virginia, the United States [39]. Based on an ecological niche model framework, they predicted that the warm weather during June and August is more suitable for the organism than the cold weather during December and March [39]. In Thailand, Supabandhu et al. successfully isolated 59 *P. insidiosum* strains from 325 water samples collected from agricultural areas (i.e., rice fields, irrigation channels, and water reservoirs) in northern Thailand [35]. They reported the isolate-per-sample (IPS) value of 59/325 or 0.18. The current study reported the IPS value of 27/500 or 0.05, which was calculated based on the *P. insidiosum*-positive samples collected from urban areas (i.e., zoo and public parks; 7 isolates per 300 samples) and agricultural areas (i.e., ponds and rice fields; 20 isolates per 200 samples) in central and southern Thailand (Table 1). The IPS value of Supabandhu et al. (0.18) was 3.4-fold higher than that of our study (0.05). In our study, the IPS value of agricultural areas (20/200 or 0.10) was 5-time higher than that of urban areas (7/300 or 0.02). *P. insidiosum* may be more prevalent in the northern part than the other parts of Thailand. On the other hand, the low prevalence may due to sampling biases, as 48% of the water samples were collected from the urban areas. Taken together, we learned that: (i) *P. insidiosum* is widespread in Thailand (and perhaps in neighborhood countries where cases are not yet reported); (ii) the organism presents in the crowded city, i.e., Bangkok; and (iii) the pathogen is more prevalent in the agricultural habitats. The higher prevalence of *P. insidiosum* in the agricultural areas was consistent with the fact that the majority of Thai patients with pythiosis were farmers living all over the country [2,35]. An individual who exposes to the ecological niche of *P. insidiosum* could become at risk of the infection. 

*P. insidiosum* is classified into 3 genotypes, in association with its geographic origins (i.e., Clade-I genotype in Americas, Clade-II genotype in Asia and Australia, and Clade-III genotype in Thailand) [52,53]. The multiplex PCR has the ability to not only detect *P. insidiosum*, but also genotype this organism into Clade-I, -II, or -III strains, simply based on size and number of the amplicons [23]. This amplification technique correctly assigned 27 water-isolated *P. insidiosum* strains into Clade-II (*n* = 19) and Clade-III (*n* = 8) genotypes, which were in agreement with the phylogenetic findings (Table 2; Figure 3). Biodiversity of the Thai water-isolated strains of *P. insidiosum* (*n* = 26; Table 2), in relation to the human and animal strains from different geographic areas (*n* = 22; Table 3), was assessed by phylogenetic and proteomic approaches. Using the rDNA-*cox*1-*cox*2 concatenated sequences, *P. insidiosum* can be grouped into 3 phylogenetic clades, as expected (Figure 3). The Thai water-isolated strains were restricted to only Clade-II and Clade-III (Table 2; Figure 3). This finding suggests that the major circulating strains of *P. insidiosum* in the Thai environment are the Clade-II and Clade-III genotypes, which are the typical genotypes of the pathogen isolated from all Thai patients [23,53]. Until 2020, a clade A_th_ (equivalent to Clade-I) strain of *P. insidiosum* was isolated from the first dog with pythiosis in Thailand [54]. Such information suggests that the Clade-I strains might also circulate in Thailand, but to a much lesser extent than the Clade-II and -III strains.

We initially explored the proteome-based biodiversity of the *P. insidiosum* isolated from water (*n* = 10), humans (*n* = 6), and animals (*n* = 3) (Table 2 and Table 3). Unlike the phylogenetic approach, the mass spectrometry-derived dendrogram divided these isolates into only 2 groups: proteotype-A (comprising Clade-I and -II genotypes) and proteotype-B (comprising only Clade-III genotypes) (Figure 5). Hence, the proteomic method exhibited less discrimination power for bio-diversifying *P. insidiosum* than the phylogenetic approach. Nine of the water-isolated strains were from the same sample collection site (Rice Field#3) and can be grouped into 2 subpopulations: proteotype-A/genotype Clade-II (*n* = 4) and proteotype-B/genotype Clade-III (*n* = 5) (Table 2; Figure 5). The proteomic (Figure 5) and phylogenetic (Figure 3) analyses demonstrated the marked biodiversity of the *P. insidiosum* subpopulation inhabiting a local environment.

In conclusion, we successfully isolated *P. insidiosum* from the urban and rural areas (including the city of Bangkok), using the hair-baiting technique. The identity of the organism was confirmed by multiplex PCR, DNA barcoding, and proteomic analysis. The combination of rDNA and *cox*2 barcodes showed superior performance for the identification of *P. insidiosum*, while the *cox*1 barcode cannot assign a species to some strains due to the lack of a comprehensive dataset in GenBank. Proteomic and phylogenetic analyses revealed subpopulations and biodiversity (i.e., proteotype-A/genotype Clade-II and proteotype-B/genotype Clade-III) of the water-isolated *P. insidiosum* strains in a local area. *P. insidiosum* is ubiquitous in Thailand and only the Clade-II and Clade-III genotypes (the typical genotypes that infect Thai patients) circulate in the environment (i.e., rice fields and ponds). Better understanding the ecological niches of *P. insidiosum* can lead to a proper measure to reduce the exposure of an individual at risk to the pathogen, and thus prevent pythiosis.

## Figures and Tables

**Table 1:** Water sampling locations in central and southern Thailand. The global positioning system (GPS) coordinates, type of water collection sites, number of sites and water samples, and identified organisms are shown in the table.

Province (Province ID; Number of *P. insidiosum* Isolates)	Sampling Locations		Type of Water Collection Site	Number of Sites	Collected Samples	Number of Culture-Positive Samples (Species; % of Collected Samples)		
	Code	GPS Coordinate				*P. insidiosum*	Other *Pythium* Species	Other Organisms
Bangkok (BK; *n* = 7)	BK01	13.770966, 100.516366	Pond (zoo)	10	50	2 (4%)	1 (*Pythium* sp.; 2%)	-
	BK02	13.854501, 100.859078	Pond (zoo)	3	15	-	-	-
	BK03	13.807507, 100.555620	Pond (public park)	8	40	-	-	-
	BK04	13.929843, 100.568340	Pond (public park)	4	20	1 (5%)	-	1 (unclassified; 5%)
	BK05	13.787263, 100.674502	Pond (public park)	3	15	-	-	-
	BK06	13.731485, 100.541550	Pond (public park)	4	20	-	-	-
	BK07	13.652241, 100.491406	Pond (public park)	4	20	-	-	-
	BK08	13.770022, 100.494723	Pond (public park)	3	15	-	-	-
	BK09	13.686055, 100.662055	Pond (public park)	7	35	4 (11%)	6 (*P. catenulatum*; 17%), 1 (*Pythium* sp.; 3%)	-
	BK10	13.744389, 100.352753	Pond (public park)	2	10	-	-	-
Chonburi (CB; *n* = 0)	CB01	13.257211, 101.151760	Pond, Irrigation channel	3	15	-	3 (*P. catenulatum*; 20%)	1 (unclassified; 7%)
Chachoengsao (CS; *n* = 6)	CS01	13.668917, 101.191042	Pond	4	20	1 (5%)	1 (*P. rhizo-oryzae*; 5%), 1 (*Pythium* sp.; 5%)	-
	CS02	13.652780, 101.161328	Pond	2	10	-	1 (*P. rhizo-oryzae*; 10%)	-
	CS03	13.606459, 101.232437	Rice field	2	10	5 (50%)	-	-
Nakhon Pathom (NP; *n* = 0)	NP01	14.020776, 99.973345	Pond	12	60	-	6 (*P. catenulatum*; 10%)	1 (*Sclerotium hydrophilum*; 2%), 1 (*Mucor amphibiorum*; 2%)
Kanchanaburi (KB; *n* = 11)	KB01	14.138575, 99.324756	Pond	5	25	1 (4%)	-	-
	KB02	13.978799, 99.659559	Pond	1	5	-	1 (*P. catenulatum*; 20%)	2 (unclassified; 40%)
	KB03	14.027293, 99.791957	Pond, Rice field	5	25	10 (40%)	1 (*Pythium* sp.; 4%)	-
Ratchaburi (RB; *n* = 1)	RB01	13.806338, 99.689354	Pond	6	30	1 (3%)	1 (*P. rhizo-oryzae*; 3%)	-
	RB02	13.571397, 99.774272	Pond	3	15	-	4 (*P. rhizo-oryzae*; 27%), 1 (*P. inflatum;* 7%)	1 (*Pezizomycetes* sp.; 7%)
	RB03	13.514250, 99.715126	Pond	2	10	-	1 (*P. rhizo-oryzae*; 10%)	-
	RB04	13.507072, 99.840394	Pond	2	10	-	-	-
Trang (TG; *n* = 2)	TG01	07.534068, 99.618507	Pond, Rice field	5	25	2 (8%)	1 (*Pythium* sp.; 4%)	-
Total (*n* = 27)	-	-	-	100	500	27 (5.4%)	30 (6.0%)	7 (1.4%)

**Table 2:** Twenty-seven strains of *P. insidiosum* successfully isolated from 5 provinces in Thailand. Sampling locations (with GPS coordinates), water sources, strain IDs, sequence homology analyses, and species-level identification and biotyping (based on DNA barcodes, multiplex PCR, and mass spectrometry) are summarized in the Table.

Province (*P. insidiosum* Isolates)	Sampling Locations		Water Source (Collection Site)	Strain ID	DNA Barcodes						Genotype (Clade)		MALDI-TOF MS	
	Code	GPS Coordinate			rDNA		*cox*1		*cox*2		DNA Barcode	Multiplex PCR	Score	Proteotype
					Accession	% Identity	Accession	% Identity	Accession	% Identity				
Bangkok (*n* = 7)	BK01	13.770966, 100.516366	Pond#1 (zoo)	BKDZ01	LC556017	99.9	LC547937	94.3	LC549516	100.0	III	III	-	-
			Pond#2 (zoo)	BKDZ02	LC556018	98.0	LC547938	94.1	LC549517	100.0	III	III	2.200	B
	BK04	13.929843, 100.568340	Pond#3 (park)	RT02	LC556073	99.9	LC547929	99.7	LC549523	100.0	II	II	-	-
	BK09	13.686055, 100.662055	Pond#4 (park)	RM9-02	LC556063	99.5	LC547930	100.0	LC549519	100.0	II	II	-	-
				RM9-03	LC556064	99.8	LC547931	100.0	LC549520	100.0	II	II	-	-
				RM9-04	LC556065	99.8	LC547932	100.0	LC549521	100.0	II	II	-	-
				RM9-05	LC556066	99.4	LC547933	100.0	LC549522	100.0	II	II	-	-
Chachoengsao (*n* = 6)	CS01	13.668917, 101.191042	Pond#5	CCS01	LC556020	99.9	LC547923	100.0	LC549500	100.0	II	II	-	-
	CS03	13.606459, 101.232437	Rice Field#1	CCS03	LC556022	99.8	LC547924	100.0	LC549501	100.0	II	II	-	-
				CCS04	LC556023	100.0	LC547925	100.0	LC549502	100.0	II	II	-	-
				CCS05	LC556024	100.0	LC547926	100.0	LC549503	100.0	II	II	-	-
			Rice Field#2	CCS07	LC556025	99.9	LC547927	100.0	LC549504	100.0	II	II	-	-
				CCS08	LC556026	99.9	LC547928	100.0	LC549505	100.0	II	II	-	-
Kanchanaburi (*n* = 11)	KB01	14.138575, 99.324756	Pond#6	KCB12	LC556043	99.4	-	-	-	-	-	II	-	-
	KB03	14.027293, 99.791957	Rice Field#3	KCB01	LC556032	99.8	LC547939	94.4	LC549506	100.0	III	III	2.490	B
				KCB02	LC556033	99.9	LC547919	99.3	LC549507	100.0	II	II	2.274	A
				KCB03	LC556034	99.7	LC547940	94.3	LC549508	100.0	III	III	2.151	B
				KCB04	LC556035	99.4	LC547941	94.4	LC549509	100.0	III	III	2.187	B
				KCB05	LC556036	99.9	LC547920	99.3	LC549510	100.0	II	II	2.149	A
				KCB06	LC556037	99.7	LC547942	94.4	LC549511	100.0	III	III	-	-
				KCB07	LC556038	100.0	LC547921	99.3	LC549512	100.0	II	II	2.196	A
				KCB08	LC556039	99.9	LC547943	94.4	LC549513	100.0	III	III	2.400	B
				KCB09	LC556040	99.8	LC547944	94.4	LC549514	100.0	III	III	2.187	B
				KCB10	LC556041	99.4	LC547922	99.3	LC549515	100.0	II	II	2.200	A
Ratchaburi (*n* = 1)	RB01	13.806338, 99.689354	Pond#7	RCB04	LC556056	99.6	LC547936	99.3	LC549518	100.0	II	II	-	-
Trang (*n* = 2)	TG01	07.534068, 99.618507	Rice Field#4	TRG02	LC556075	99.9	LC547934	100.0	LC549524	100.0	II	II	-	-
			Pond#8	TRG03	LC556076	99.9	LC547935	100.0	LC549525	100.0	II	II	-	-

**Table 3:** Twenty-two clinical strains of *P. insidiosum* used for proteomic and phylogenetic analyses in this study. The table contains strain IDs, reference IDs, affected hosts (i.e., humans or animals), country of origins, mass spectrometry-based prototypes, and GenBank accessions (i.e., rDNA, *cox*1, and *cox*2).

Genotype	Strain ID	Reference ID	Source	Country	Proteotype	GenBank Accession		
						rDNA	*cox*1	*cox*2
Clade-I (*n* = 7)	Pi08	CBS 580.85	Horse	Costa Rica	A	AB898107	LC553008	LC553029
	Pi10	ATCC 200269	Human	USA	A	AB898108	LC553003	LC553024
	Pi59	EQ02	Horse	Brazil	not done	LC550290	LC553010	LC553031
	Pi60	EQ04	Horse	Brazil	not done	LC550291	LC553011	LC553032
	Pi62	EQ06	Horse	Brazil	not done	LC550293	LC553012	LC553034
	Pi07	CBS 573.85	Horse	Costa Rica	not done	AB971180	LC553007	LC553028
	Pi74	KU40017.3	Dog	Thailand	not done	MT459311	LC553013	LC553035
Clade-II (*n* = 9)	Pi23	MCC10	Human	Thailand	not done	AB898115	LC553019	LC553038
	Pi25	P19	Human	Thailand	not done	AB898116	LC553020	LC553039
	Pi29	SIMI 1126-46	Human	Thailand	not done	LC199882	LC553016	LC553040
	Pi32	P34	Human	Thailand	not done	AB898121	LC553017	LC553041
	Pi35	Pi-S	Human	Thailand	A	AB898124	BAS30577	BAS30578
	Pi36	ATCC 64221	Horse	Australia	A	LC199883	LC553005	LC553026
	Pi37	ATCC 28251	Horse	Papua New Guinea	not done	LC199884	LC553004	LC553025
	Pi40	CBS 777.84	Mosquito	India	A	LC199886	LC553009	LC553030
	Pi53	P39	Horse	Thailand	not done	LC199889	LC553018	LC553042
Clade-III (*n* = 6)	Pi44	MCC 17	Human	Thailand	not done	AB971185	LC553015	LC553037
	Pi45	MCC 13	Human	Thailand	B	AB971186	LC553014	LC553036
	Pi46	SIMI 3306-44	Human	Thailand	not done	AB971187	LC553022	LC553045
	Pi47	SIMI 2921-45	Human	Thailand	B	AB971188	LC553021	LC553044
	Pi49	SIMI 7695-48	Human	Thailand	B	AB898127	LC553023	LC553046
	Pi50	ATCC 90586	Human	USA	B	AB971190	LC553006	LC553027

**Table 4:** A list of 64 culture-positive samples that show a white-to-colorless, non-sporulation, submerged colony, which is compatible with the gross morphology of *P. insidiosum*. The table includes sample collection locations and sites, organism identities, strain IDs, percent sequence identities, DNA barcode-based genotypes (i.e., Clade I, II, and III), multiplex PCR results (i.e., positive or negative; Clade I, II, and III), and GenBank accessions of DNA barcodes (i.e., rDNA, *cox*1, and *cox*2).

No	Sample Collection Locations (Province)	Water Collection Sites	Isolated Organisms	Strain ID	% Identity	DNA Barcode-Based Genotype (Clade)	Multiplex PCR Results (Clade)	GenBank Accessions		
								rDNA	*cox*1	*cox*2
1	Bangkok	Zoo	*Pythium insidiosum*	BKDZ01	99.9	III	Positive (III)	LC556017	LC547937	LC549516
2	Bangkok	Zoo	*Pythium insidiosum*	BKDZ02	98.0	III	Positive (III)	LC556018	LC547938	LC549517
3	Bangkok	Zoo	*Pythium* species	BKDZ03	99.1	-	Negative	LC556019	-	-
4	Bangkok	Public park	*Pythium* species	RM9-01	95.9	-	Negative	LC556062	-	-
5	Bangkok	Public park	*Pythium insidiosum*	RM9-02	99.5	II	Positive (II)	LC556063	LC547930	LC549519
6	Bangkok	Public park	*Pythium insidiosum*	RM9-03	99.8	II	Positive (II)	LC556064	LC547931	LC549520
7	Bangkok	Public park	*Pythium insidiosum*	RM9-04	99.8	II	Positive (II)	LC556065	LC547932	LC549521
8	Bangkok	Public park	*Pythium insidiosum*	RM9-05	99.4	II	Positive (II)	LC556066	LC547933	LC549522
9	Bangkok	Public park	*Pythium catenulatum*	RM9-06	99.0	-	Negative	LC556067	LC553640	LC553642
10	Bangkok	Public park	*Pythium catenulatum*	RM9-07	99.9	-	Negative	LC556068	-	-
11	Bangkok	Public park	*Pythium catenulatum*	RM9-08	99.6	-	Negative	LC556069	-	-
12	Bangkok	Public park	*Pythium catenulatum*	RM9-09	99.7	-	Negative	LC556070	-	-
13	Bangkok	Public park	*Pythium catenulatum*	RM9-10	99.5	-	Negative	LC556071	-	-
14	Bangkok	Public park	*Pythium catenulatum*	RM9-11	98.6	-	Negative	LC556072	-	-
15	Bangkok	Public park	*Pythium insidiosum*	RT02	99.9	II	Positive (II)	LC556073	LC547929	LC549523
16	Bangkok	Public park	Unclassified	RT01	-	-	Negative	-	-	-
17	Chachangsao	Pond	*Pythium insidiosum*	CCS01	99.9	II	Positive (II)	LC556020	LC547923	LC549500
18	Chachangsao	Pond	*Pythium* species	CCS02	99.5	-	Negative	LC556021	-	-
19	Chachangsao	Pond	*Pythium insidiosum*	CCS03	99.8	II	Positive (II)	LC556022	LC547924	LC549501
20	Chachangsao	Pond	*Pythium insidiosum*	CCS04	100.0	II	Positive (II)	LC556023	LC547925	LC549502
21	Chachangsao	Pond	*Pythium insidiosum*	CCS05	100.0	II	Positive (II)	LC556024	LC547926	LC549503
22	Chachangsao	Pond	*Pythium insidiosum*	CCS07	99.9	II	Positive (II)	LC556025	LC547927	LC549504
23	Chachangsao	Pond	*Pythium insidiosum*	CCS08	99.9	II	Positive (II)	LC556026	LC547928	LC549505
24	Chachangsao	Pond	*Pythium rhizo-oryzae*	CCS13	99.8	-	Negative	LC556031	-	-
25	Chonburi	Pond	Unclassified	CCS06	-	-	Negative	-	-	-
26	Chonburi	Pond	*Pythium rhizo-oryzae*	CCS09	99.6	-	Negative	LC556027	-	-
27	Chonburi	Irrigation channel	*Pythium catenulatum*	CCS10	99.8	-	Negative	LC556028	-	-
28	Chonburi	Pond	*Pythium catenulatum*	CCS11	99.8	-	Negative	LC556029	-	-
29	Chonburi	Pond	*Pythium catenulatum*	CCS12	99.9	-	Negative	LC556030	-	-
30	Kanchanaburi	Rice field	*Pythium insidiosum*	KCB01	99.8	III	Positive (III)	LC556032	LC547939	LC549506
31	Kanchanaburi	Rice field	*Pythium insidiosum*	KCB02	99.9	II	Positive (II)	LC556033	LC547919	LC549507
32	Kanchanaburi	Rice field	*Pythium insidiosum*	KCB03	99.7	III	Positive (III)	LC556034	LC547940	LC549508
33	Kanchanaburi	Rice field	*Pythium insidiosum*	KCB04	99.4	III	Positive (III)	LC556035	LC547941	LC549509
34	Kanchanaburi	Rice field	*Pythium insidiosum*	KCB05	99.9	II	Positive (II)	LC556036	LC547920	LC549510
35	Kanchanaburi	Rice field	*Pythium insidiosum*	KCB06	99.7	III	Positive (III)	LC556037	LC547942	LC549511
36	Kanchanaburi	Rice field	*Pythium insidiosum*	KCB07	100.0	II	Positive (II)	LC556038	LC547921	LC549512
37	Kanchanaburi	Rice field	*Pythium insidiosum*	KCB08	99.9	III	Positive (III)	LC556039	LC547943	LC549513
38	Kanchanaburi	Rice field	*Pythium insidiosum*	KCB09	99.8	III	Positive (III)	LC556040	LC547944	LC549514
39	Kanchanaburi	Rice field	*Pythium insidiosum*	KCB10	99.4	II	Positive (II)	LC556041	LC547922	LC549515
40	Kanchanaburi	Rice field	*Pythium* species	KCB11	99.6	-	Negative	LC556042	-	-
41	Kanchanaburi	Rice field	*Pythium insidiosum*	KCB12	99.4	-	Positive (II)	LC556043	not done	not done
42	Kanchanaburi	Rice field	Unclassified	KCB13	-	-	Negative	-	-	-
43	Kanchanaburi	Rice field	Unclassified	KCB14	-	-	Negative	-	-	-
44	Kanchanaburi	Rice field	*Pythium catenulatum*	KCB15	99.0	-	Negative	LC556044	-	-
45	Nakhon Pathom	Pond	*Pythium catenulatum*	KPS01	99.8	-	Negative	LC556045	-	-
46	Nakhon Pathom	Pond	*Pythium catenulatum*	KPS02	99.6	-	Negative	LC556046	-	-
47	Nakhon Pathom	Pond	*Mucor amphibiorum*	KPS03	95.4	-	Negative	LC556047	-	-
48	Nakhon Pathom	Pond	*Pythium catenulatum*	KPS04	99.6	-	Negative	LC556048	-	-
49	Nakhon Pathom	Pond	*Pythium catenulatum*	KPS05	99.8	-	Negative	LC556049	-	-
50	Nakhon Pathom	Pond	*Sclerotium hydrophilum*	KPS06	90.5	-	Negative	LC556050	-	-
51	Nakhon Pathom	Pond	*Pythium catenulatum*	KPS07	99.8	-	Negative	LC556051	-	-
52	Nakhon Pathom	Pond	*Pythium catenulatum*	KPS08	99.8	-	Negative	LC556052	-	-
53	Ratchaburi	Pond	*Pythium rhizo-oryzae*	RCB01	99.9	-	Negative	LC556053	LC553639	LC553641
54	Ratchaburi	Pond	*Pythium rhizo-oryzae*	RCB02	99.6	-	Negative	LC556054	-	-
55	Ratchaburi	Pond	*Pythium rhizo-oryzae*	RCB03	99.9	-	Negative	LC556055	-	-
56	Ratchaburi	Pond	*Pythium insidiosum*	RCB04	99.6	II	Positive (II)	LC556056	LC547936	LC549518
57	Ratchaburi	Pond	*Pythium inflatum*	RCB05	100.0	-	Negative	LC556057	-	-
58	Ratchaburi	Pond	*Pythium rhizo-oryzae*	RCB06	99.4	-	Negative	LC556058	-	-
59	Ratchaburi	Pond	*Pezizomycetes* species	RCB07	99.8	-	Negative	LC556059	-	-
60	Ratchaburi	Pond	*Pythium rhizo-oryzae*	RCB08	99.8	-	Negative	LC556060	-	-
61	Ratchaburi	Pond	*Pythium rhizo-oryzae*	RCB09	99.6	-	Negative	LC556061	-	-
62	Trang	Rice field	*Pythium* species	TRG01	95.9	-	Negative	LC556074	-	-
63	Trang	Rice field	*Pythium insidiosum*	TRG02	99.9	II	Positive (II)	LC556075	LC547934	LC549524
64	Trang	Ponds	*Pythium insidiosum*	TRG03	99.9	II	Positive (II)	LC556076	LC547935	LC549525
